# Releasing the GENIE and Other Tips for Life Sciences Teachers

**DOI:** 10.15694/mep.2017.000109

**Published:** 2017-06-21

**Authors:** Barbara A. Jennings, Harpreet Bassi, Yoon K. Loke

**Affiliations:** 1 UEA

**Keywords:** pharmacology, genetics, technology enhanced learning, systems based curriculum

## Abstract

This article was migrated. The article was marked as recommended.

This paper describes practical tips and tools for delivering life sciences teaching, it is based on the authors’ experiences at Norwich Medical School (NMS), which opened in 2002 at the University of East Anglia. The inaugural medical curriculum, including its ethos and how it is perceived by staff and students, have all been described previously.⁽¹⁻³⁾ Our 5 year medical degree at NMS is a modular, systems-based programme that integrates theory and clinical practice from the beginning. We use problem based learning (PBL) but teaching is also delivered via clinical placement; structured and simulated patient teaching; and via formal lectures and workshops. Core social science themes and life science themes run longitudinally through the course and spiral delivery of learning outcomes allows students to re-visit our themes, and particular topics, with increasing complexity.

We offer ten tips here, about delivering the curricula for genetics, pharmacology, and prescribing. One challenge that we face, with a modular degree programme, is that we meet our students intermittently and throughout their years of study, and our science curriculum has been developed in response to this.

## Ten Tips

The suggestions that we offer map to some of the roles presented by Harden and Crosby in their appraisal of the *good teacher* in medical education ^(4)^; particularly the *information provider* and *the resource material creator.* Our ten tips explicitly discuss our own specialities but we think the principles are generalizable to the delivery of other life sciences too.

1. We might expect that modern educational theory, which encourages a learner-centred rather than teacher-centred approach, would have sounded the death knell for *The Lecture.* However, most medical school curricula still deliver a lot of 60 minute lecture slots. There are some very practical reasons for this, including the available teaching-space in buildings and the ease of timetabling uniform blocks of teaching. But there may be some compelling social reasons too, lectures allow for the congregation of peers, and the sharing of some inspiring clinical and research stories. Our tip for didactic lectures is to use them sparingly and reserve them for some well-considered storytelling that will engage your learners emotionally and intellectually.
2. Of course, not all lectures have to be didactic; and wherever possible do flip-your-classroom ^(5)^. Encourage students to be responsible for their learning by providing factual material before the class and then spending teaching-time engaged with interactive learning materials, exercises, problem-solving, and discussion. However, our advice for a sustainable curriculum is to use flipped-lectures sparingly too. For the genetics curriculum, we only use this approach for complex family history and data interpretation workshops. The challenges to overcome for flipped lecturing include the need for a very clear line of communication between teacher and students about expectations before the teaching session; coupled with adequate staffing to make workshops and the problem-solving productive (i.e. resulting in measurable learning gain). The pre-reading or study before the classroom-time can be delivered as a simple PDF or through more complex forms of technology enhanced learning (TEL); please see tip number 10 below.
3. Defining a core (mandatory) life sciences curriculum is a challenge. As researchers and teachers we are very enthusiastic about our own areas of medical science but we need to present a balanced and reasonable curriculum for our busy learners. One tip is to benchmark your life sciences curriculum against indicative specialist national curricula, but also ask generalists within your organisation about how much a graduate really has to know, and adjust expectations accordingly. In pharmacology and genetics there have been national initiatives and research efforts to define the core undergraduate curriculum for some time ^(6-9)^ and they have led to the development of useful linked resource libraries that can be shared. Moreover, pharmacological knowledge and associated skills in interpreting drug information are essential elements within the UK National Prescribing Safety Assessment (PSA), thus shaping the curriculum to ensure that students become competent prescribers.
4. It is impossible to fit expanding fields of science coupled with individual research opportunities, into finite and fixed timetable blocks. But for the genetics and pharmacology strands at NMS, we have found that we can still offer opportunities for learners who seek advanced knowledge or research attachments by using student selected components (SSCs). SSCs allow a more fluid approach to teaching which permits personalised-learning and the production of research outputs. In NMS SSCs are used to help students to develop a range of research skills. ^(10)^
5. To encourage their engagement with the wider scientific community, you can introduce your learners to leading scientists in your field and to the journals and learned societies that you all subscribe to by following them via social media platforms such as twitter. Twitter accounts can then be linked to your learning resources including PowerPoint files.
6. A curriculum that is modular creates a challenge if we want to provide an integrated and holistic experience for our medical students. Our tip for those who lead life science teaching strands is to work closely with module and other curriculum leads to ensure that your science curriculum reaches beyond your individual classes. For example, review and help to edit handbooks to ensure there is accurate and inspiring specialist information to serve as trigger material in PBL scenarios and in structured clinical teaching.
7. On the subject of PBL scenarios, we find that well-structured clinical simulations bring life sciences to “life”. To the student, the joy and value of learning science is most apparent when it can be used to explain clinical phenomena in human beings. It’s all too easy for students to adopt a pattern-recognition or algorithmic approach: for example, swelling of the big toe and raised uric acid in the patient indicates the diagnosis of gout, which should be treated in the long-term by the drug allopurinol. With this example, challenge the students to explain how and why gout causes painful swollen joints, and the biochemical pathway that leads to an abnormal elevation in uric acid. The science will also help them determine why allopurinol is more suitable as a long-term treatment than ibuprofen. Here, the empiricism of medicine needs to be underpinned by focused, relevant scientific teaching. The science does not necessarily need to be esoteric, cutting edge, or complex - if the explanation for the clinical phenomenon is simple, so be it, that’s all the student needs to know.
8. The research progress for some fields of science is particularly rapid and this presents problems for learners tasked with understanding them and with applying evidence based medicine. For human genomics and pharmacogenetics we see the challenges that undergraduate and postgraduate students face as they navigate their way through the literature and data. One role that teachers have is to effectively curate and annotate information. Two years ago we designed a web microsite hosted at Norwich Medical School to help with this task; its name is *GENIE,* which is an abbreviation of genetic-information-and-education (please see www.uea.ac.uk/cpd/genie). We sign-post and explain educational and research resources for human genomics; some will be useful to first year undergraduates but others will be for the expert research-student, engaged with *in silico* experiments. The scope of the microsite is purposefully wide, complementing roles as an undergraduate teacher, research supervisor, and facilitator of CPD for qualified doctors. The microsite is a developing resource that requires updates and checks for broken links, and so is not without cost. But all teachers spend time each year organising their new reading lists and resources; and by directing users to a single site this process has become more efficient for the theme lead, and more transparent for students. An added bonus is that by creating open, web-based resources, the impact of our teaching can extend beyond learners at a single institution.
9. Another component of the teacher’s toolkit in 2017 is the educational app. The introduction of the UK PSA helped all medical schools to review their students’ performance across eight stations of the exam, and calculation skills were flagged up as an area for improvement, both locally and nationally. At NMS we responded to this by providing drug calculation exercises on our virtual learning environment (VLE) for all medical students to use, but statistics collected from the VLE revealed both poor uptake and poor skill-retention. Focus groups with students were also used to explore poor student engagement. The academic lead for prescribing has created the tool called Matha *Medic* in response to this challenge; it was the first drug calculations app designed for medical students. This educational resource is available for free download via two app-store sites (please see https://play.google.com/store/apps/details?id=com.uea.mathamedic&hl=en and https://itunes.apple.com/gb/app/mathamedic/id992471981?mt=8). Its popular features include an intuitive user interface and interactive online support, and with over 400 downloads Matha *Medic* is proving to be a hit.
10. Both genetics and pharmacology curricula at NMS use a blended learning approach; complementing face-to-face teaching with online learning resources. The use of TEL has many advantages. It can facilitate distance and asynchronous learning when students are in dispersed placements; it promotes independent study and time for students to engage in critical thinking; it solves accommodation and timetabling challenges; and facilitates integration between disciplines. We have developed online lessons about pharmacogenetics and stratified medicine that are delivered within a VLE; we have used the Blackboard VLE and Moodle for online delivery at NMS. Online lessons allow students to revisit and integrate fundamental principles of pharmacology and genetics as well as an evidence-based appraisal of current translational research. For all TEL initiatives, our main tip is to remember that educational principles are more important than technological gimmicks, so trigger interest with clinical vignettes; promote active learning through discussion boards and the use of regular assignments; and do use step-wise delivery. Having stressed the pedagogy, the creative aspects of TEL are fun for the teacher too, consider using whiteboard animations and interviews with research colleagues for impact and interest.

Harden and Crosby defined the good teacher very simply as, “ *a teacher who helped the student to learn*”. ^(4)^ These practical tips were written to share our ideas and experiences about popular and helpful teaching strategies used at NMS.

## Take Home Messages

We present ten practical tips and tools that have proved useful to teachers of genetics, pharmacology, and prescribing at a UK medical school.

We think the guidance and suggestions are particularly applicable to the teaching of life sciences in a systems-based, modular, degree programme.

## Notes On Contributors

Dr Barbara Jennings joined Norwich Medical School as part of the inaugural team when it was established in 2002, and is a Senior Lecturer in the Medical Education department. She is the academic lead for the genetics curriculum, and for faculty continuous-professional-development. Barbara has a background in cancer research and in clinical molecular diagnostics. Her research spans cancer genetics, genetic epidemiology and pharmacogenetics. Barbara is a member of the MedEdPublish editorial board and is Senior Fellow of the Higher Education Academy.

Harpreet Kaur Bassi is a Specialist Clinical Pharmacist & Senior Medical Lecturer at Norwich Medical School. Harpreet is theme lead for prescribing (MBBS) and prescribing education lead for foundation Doctors. Primary research interests in medication safety & optimisation through inter-professional education & Technology enhanced learning. Developer of Matha *Medic*: 1 ^st^ drug calculations App for medical students, Harpreet is also nominated Digital technology lead for ASME TEL SIG. Fellow of the Higher Education Academy, Senior Author for the Prescribing Skills Assessment and an Executive for the Centre for Pharmacy Postgraduate Education operations board.

Dr Yoon K Loke is Professor of Medicine and Pharmacology at the University of East Anglia, and is a practising clinician as well as keen researcher and teacher. His current work is focused on data sources and risk of bias in the evaluation of adverse effects of drug therapy. Dr Loke is Chair of the Joint Speciality Committee for Clinical Pharmacology & Therapeutics, Royal College of Physicians London.

## Declarations

Barbara Jennings is one of the guest Theme Editors for the AMEE MedEdPublish themed issue, Life Sciences in an Integrated Curriculum and is a member of the AMEE MedEdPublish Editorial Board.
