# Supplementary material for: Releasing the GENIE and Other Tips for Life Sciences Teachers
Source: MedEdPublish (2016). 2017 Jun 21;6:109. [Version 1] doi: 10.15694/mep.2017.000109 (PMC10885265; doi:10.15694/mep.2017.000109)
Supplement: Peer review history

# Reviewer report: Reviewer response for version 1

**DOI:** 10.21956/mep.19263.r27990

**Published:** 2019-04-03

**Authors:** Ken Masters

**Affiliations:** 1 Sultan Qaboos University

This review has been migrated. The reviewer awarded 3 stars out of 5 An interesting read, although possibly more useful for the novice than the experienced teacher; nevertheless, the paper does have value. In a tips paper like this, however, it would have been more valuable if the authors had briefly identified each tip in a single statement, and then expanded upon it. As it is written, the reader had to read through quite a bit of material before finding the tip. (e.g. in the first tip, the tip is found only in the final sentence; others are not buried so far down, but few are the first sentence of the paragraph, which where they would have the greatest impact.)Similarly, it would have been useful if the tips could have been summarised briefly in the Abstract; again, to enable quick reading so that readers can gloss over those they already know, and quickly find the new ideas.So, some useful insights, but, with a few changes, could be structured in a more reader-friendly manner.

Reviewer Expertise:

NA

No decision status is available

# Reviewer report: Reviewer response for version 1

**DOI:** 10.21956/mep.19263.r27991

**Published:** 2017-06-21

**Authors:** Trevor Gibbs

**Affiliations:** 1 AMEE

This review has been migrated. The reviewer awarded 4 stars out of 5 An interesting list of ten tips that pulls together appropriate actions for both the curriculum and course developer. In addition it points to some innovative activities that aid student learning.I am not sure that many faculty will learn much new from the tips but they are succinct and useful and should be read

Reviewer Expertise:

NA

No decision status is available
